# Solvent effect on the absorption and emission spectra of carbon dots: evaluation of ground and excited state dipole moment

**DOI:** 10.1186/s13065-021-00779-6

**Published:** 2021-09-25

**Authors:** Parisa Mohammad-Jafarieh, Abolfazl Akbarzadeh, Rahman Salamat-Ahangari, Mohammad Pourhassan-Moghaddam, Kazem Jamshidi-Ghaleh

**Affiliations:** 1 Department of Chemistry, Faculty of Basic Sciences, Azarbaijan Shahid Madani University, 35 km Tabriz-Maraghe Road, P. O. Box 53714161, Tabriz, Iran; 2 Department of Medical Biotechnology, Faculty of Advanced Medical Sciences, Tabriz University of Medical Sciences, Tabriz, Iran; 3 Department of Medical Nanotechnology, Faculty of Advanced Medical Sciences, Tabriz University of Medical Sciences, Tabriz, Iran; 4 Department of Physics, Faculty of Basic Sciences, Azarbaijan Shahid Madani University, 35 km Tabriz-Maraghe Road, Tabriz, Iran; 5 Department of Medical Nanotechnology, Faculty of Medical Sciences, Tabriz University of Medical Sciences, Tabriz, Iran

**Keywords:** Carbon dots, Photoluminescence, Dipole moment, Quantum yield, KAT parameters, Solvatochromic

## Abstract

**Background:**

Carbon dots (C-dots) are photoluminescent nanoparticles with less than 10 nm in size. Today, many studies are performed to exploit the photoluminescence (PL) property of carbon dots, and our focus in this study is to estimate the dipole moment of carbon dots. For reaching our aims, C-dots were synthesized and dissolved in the different solvents.

**Results:**

Carbon dots with intense photoluminescence properties have been synthesized by a one-step hydrothermal method from a carbon bio-source. In this research, we report on the effect of aprotic solvents on absorption and fluorescence spectra and dipole moments of C-dots dispersed in a range of many aprotic solvents with various polarity and dielectric constant at room temperature. The change in the value of dipole moment was estimated by using the Stokes shifts. The difference between the dipole moment of the excited state and the ground state was shown using an extended form of Lippert equations by Kawski and co-workers.

**Conclusions:**

The values found for μ_g_ = 1.077 D, and μ_e_ = 3.157 D, as well as the change in the dipole moments. The results showed that the dipole moment of the excited state is more than the ground state, indicating a high density and redistribution of electrons in the excited state. Finally, the quantum yield of C-dots in the eclectic aprotic solvents was communicated and discussed.

## Introduction

Carbon-based nanomaterials such as carbon nanotubes, fullerene and graphene have poor solubility in water and lack strong fluorescence in the visible area, limiting their applications [1]. These shortcomings can be addressed by carbon dots (C-dots) which are spherical carbon-based nanoparticles with a size of less than 10 nm [2]. C-dots are heavily fluorescent, non-blinking, water soluble, chemically stable and can be easily synthesized at low cost [3, 4]. Also, introduction of full-color fluorescent C-dots [5] is another advantage that can expand their application spectrum. C-dots was first discovered by electrophoretic purification of single-walled carbon nanotubes in 2004 [6]. In recent years, different materials and synthesis methods have been used to obtain C-dots. The synthesis approaches of C-dots can be classified into two categories: top-down and bottom-up methods including hydrothermal [7, 8], electrochemical oxidation [9], acidic oxidation [10], microwave [11, 12], and laser ablation [13].

Owing to their physicochemical properties, C-dots can take part in the chemiluminescence reaction as oxidants, emitting species, energy acceptors of chemical reaction energy or even as catalyst involving in different chemiluminescence systems [14, 15]. For the first time, Shen and et al. fabricated chemiluminescence C-dots and used it to develop new class of CL nanosensors for the imaging Reactive oxygen species [16]. Also C-dots have gained widespread attention in recent years, especially in chemical censoring [17], biosensing [18], bioimaging [19], drug delivery [20], solar cells [21], light-emitting diode (LED) [22], and electrocatalysis [23].

C-dots are easily dispersed in protic and aprotic solvents due to carboxyl, hydroxyl, and carbonyl groups. The interaction between C-dots and solvent plays an essential role in the wavelength of photoluminescence emissions. In summary, no single theory can be used for a quantitative explanation of the effects of the environment on fluorescence. Explanation of these effects depends not only on polarity considerations but also on the structure of the C-dots and the types of chemical interactions it can experience with other near molecules. Kumar et al*.* [24] report on solvent-dependent spectroscopic study of fluorescent carbon nanoparticles in organic solvents. They have found that the absorption spectra of the nanoparticles were independent of solvent nature, while their photoluminescence spectra were considerably dependent on the solvent nature. The trends observed with solvent polarity follow the theory of general solvent effects, which may give the impression that solvent polarity is the only factor to consider. Solvent–solute interaction and the trace of solvent environments are investigated by considering various solvent parameters such as hydrogen bond capability, hydrogen bond acceptability and polarization on dipole moment [25, 26]. Determining the dipole moment of electron balances is crucial because it can explain how electron distribution changes under excitation. Suppan has shown that the most acceptable method to approximate the excited dipole moment of a solute involves the simultaneous estimation of the absorption and fluorescence spectra of the solute in the range of solvent [27]. C-dot fluorescence properties are complicated by dependence on excitation wavelength [28] and solvent nature [24, 29]. Because there are functional groups on the C-dots surface, they are therefore available to solvent molecules so that strong interactions of C-dots with solvent molecules can have a significant effect on fluorescence. We use the solvatochromic method in which the amount of polarity of the base and dipole moment was calculated, followed by the investigation of their differences in a variety of aprotic solvents.

Different methods [28, 30–34] have been introduced for solvatochromic measurement of the dipole moment.

Pursuing our previous work on protic solvents [35], we aim to understand the effect of intermolecular interactions of synthesized C-dots and aprotic solvents. The focus is to study the spectral changes of C-dots in aprotic solvents by using the concept of Kamlet-Abboud-Taft’s linear solubility energy. Kamlet-Abboud-Taft’s equation is one of the most reliable methods of measuring solvent effects on dissolved C-dots. This equation applies the solvent polarity parameter result on the solute’s spectral features [36].

## Experimental

### Materials

C-dots were synthesized by hydrothermal treatment of persimmon peel. All the solvents used in this research were of the highest degree of purity available from Merck. The physical properties and polarity functions of the solvents are given in Table 1; Spectroscopic polarity parameters in various aprotic solvents are provided in Table 2.

**Table1:** Physical properties, and polarity functions in aprotic solvents

Solvents	ε	n	π^*^	α	β	QY
DMSO	47.24	1.47	1	0	0.76	0.26
Acetonitrile	36.64	1.34	0.75	0.19	0.4	0.12
DMF	38.25	1.43	0.88	0	0.69	0.17
DCM	9.1	1.42	0.82	0.13	0.1	0.12
Acetic Acid	6.15	1.37	0.64	1.12	0.45	ND
Diethyl ether	4.33	1.34	0.27	0	0.47	0.53
Dioxane	2.3	1.42	0.55	0	0.37	0.10

**Table 2:** Solvent effects on the position of absorption and fluorescence maxima of C-dots: spectroscopic polarity parameters in various aprotic solvents

Solvents	ν_a_ (kK) = 10^3^ cm^−1^	ν_f_ (kK)	ν_a_ − ν_f_	ν_a_ + ν_f_	f(ε,n)	f(ε,n) + 2 g(n)
DMSO	35.77	21.92	13.84	57.70	0.841	1.660
Acetonitrile	35.71	22.19	13.51	57.91	0.862	1.533
DMF	35.21	22.22	12.98	57.43	0.839	1.606
DCM	35.58	22.47	13.11	58.05	0.597	1.353
Acetic Acid	35.46	22.57	12.88	58.03	0.497	1.200
Diethyl ether	36.49	22.72	13.76	59.22	0.382	1.054
Dioxane	35.58	22.88	12.70	58.47	0.045	0.801

### Synthesis and characterization of C-dots

The green C-dots synthesis method, their characterization and structures, were thoroughly described in our previous work [38].

Briefly, the C-dots were synthesized from persimmon peels by hydrothermal treatment. In the first step, persimmon was cut into pieces and ground into a mixture. Then, 50 mL ultrapure water was added, and the solution was kept for 15 min under magnetic stirring, and the obtained juice was autoclaved using a Teflon lined stainless steel autoclave reactor at 120 °C for 150 min. The autoclave was allowed to cool at room temperature, and the resultant dark brown solution was centrifuged at 10,000 rpm for 20 min to separate the larger particles. In the next step, the pH of the aqueous solution was adjusted to neutral with 1 M NaOH, and the C-dots solution was filtrated with a 0.22 μm filter membrane. In the final step, the C-dots solution was further purified by dialysis against (1000MWCO) deionized water for 24 h. The powder of C-dots was obstinate by lyophilization for 48 h and stored at 4 °C until further use. The formation of C-dots with an average size of 2 nm was obtained. Together with elemental analysis by CHN-analyser and using FE-SEM, the nitrogen and carbonyl-containing functional groups on the surface of C-dots were also revealed by the FTIR method. Accordingly, for further spectroscopic analyses, a 0.1% (W/V) C-dots solution was prepared in aprotic solvents by mixing for 4 h to obtain homogenous solutions.

### Absorption and emission spectroscopy

UV–Vis absorption spectra of the C-dot solutions in different aprotic solvents were recorded by a CECIL CE7250 Spectrophotometer with a 1 cm quartz cuvette at room temperature over a wavelength range of 200–600 nm. Meanwhile, photoluminescence (PL) measurements were undertaken using a Cytation 5/Biotek/USA fluorescence spectrophotometer with excitation slit set at 1 nm pass and emission at 1 nm bandpass in 96 cell plates.

## Results and discussion

The theory of universal solvent effects provides beneficial information for consideration of solvent-dependent spectral shifts. In explaining general solvent effects, the C-dots is a dipole in a continuous medium of uniform dielectric constant.

The interactions between the solvent and C-dots affect the energy difference between the ground and excited states and the orientation polarizability of solvents. The dipole moment represents the electron distribution in a molecule with a specific structure. In combination with the reactive field around it, the dipole moment plays an essential role in the transition of a molecule because a molecule can absorb light when its dipolar moment changes.

C-dots' absorption and fluorescence emission spectra in the range of different aprotic solvents with dielectric constant and refractive index were recorded at room temperature.

**Fig. 1:**
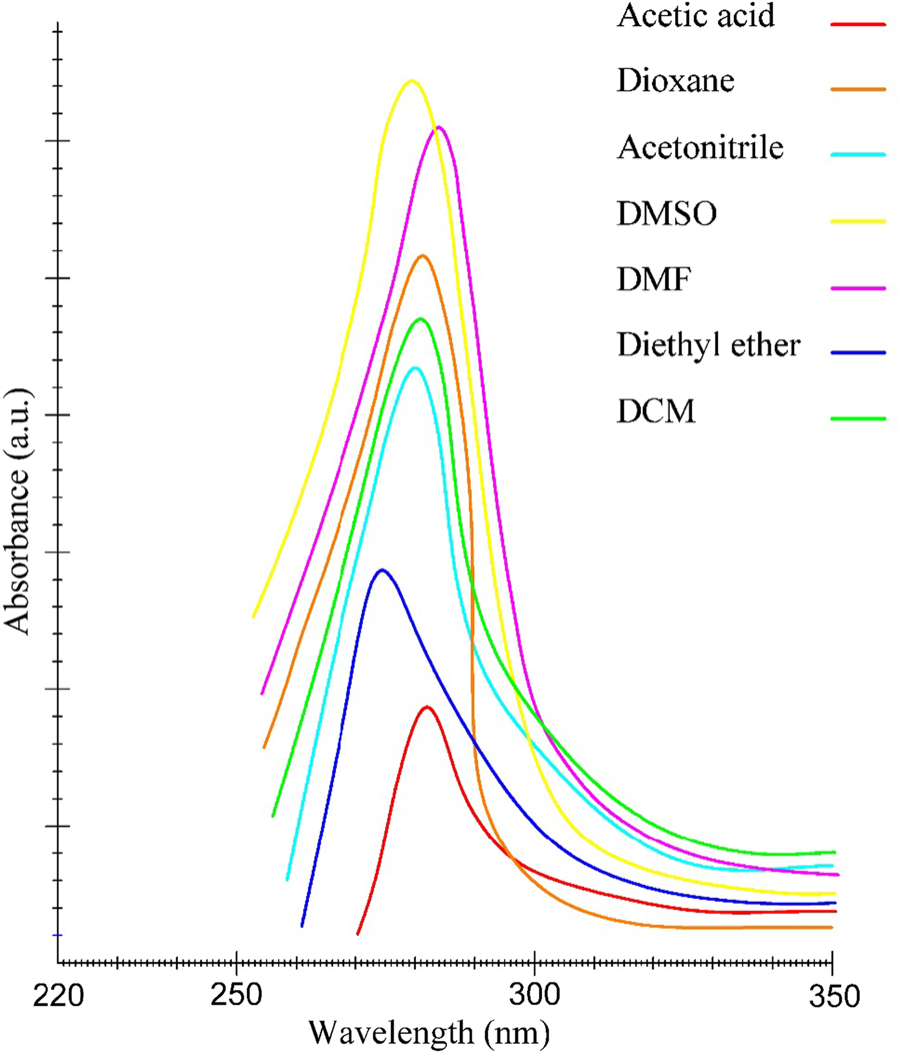
UV–vis absorption spectra of C-dots in aprotic solvents

Depending on the solvent's polarity, the aprotic solvent used in the registration of UV–Vis absorption and emission fluorescence spectra of the C-dots, depending on the solvent's polarity, influenced the positions, intensity, and shape of the solvent-C-dots complex. The UV–vis absorption spectra of the C-dots were observed in the UV region with maximum absorption at 237–256 nm and a tail extending into the visible range (Fig. 1). This is attributed to the n–π* transition of C=O band and π–π* transition of C=C band.

**Fig. 2:**
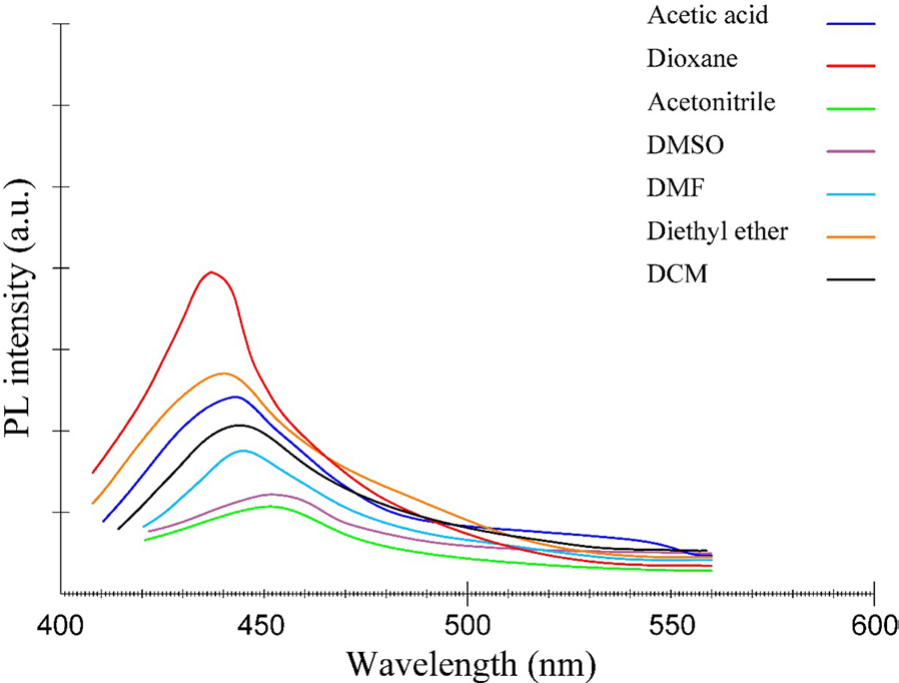
Florescence spectra of C-dots in aprotic solvents

The behavioural mechanism of PL is not yet fully understood, and our recent studies in calculating the ground and excited state dipole moment are a step forward in understanding the mechanism of this C-dots effect. One possible reason for the PL behaviour is the presence of different particle sizes of C-dots; and the different distribution of C-dots surface energy traps, the nature of the surface, and the presence of numerous functional groups on the surface of the C-dots may result in a series of emissive traps between π and π* of C–C. [37]. The results obtained from the absorption and fluorescence spectra in Fig. 1 and 2 shows that the displacement observed in the absorption and emission spectra of C-dots indicates the dependence of C-dots on solvent polarity, which means that the change of solvent polarity displacement in the emission spectra is relative to the absorption spectra.

### Estimation of the ground state and excited state dipole moments

In order to approximate the ground state and excited state dipole moments of the C-dots, spectral shifts (ῡ_A_ − ῡ_F_) and (ῡ_A_ + ῡ_F_) of fluorescence C-dots were calculated along with solvent polarity (ν_A_ and ν_F_ are the wavenumbers (cm^–1^) of the absorption and emission).

The result demonstrates that the excited dipole moment is larger during the C-dots electronic transition than the ground dipole moment, i.e. μ_e_ > μ_g._ Therefore, the dipolar solvent polarisation, the Franck–Condon excited state, is more solvated foremost to the experiential redshift in the spectrum.

To a first estimate, this energy difference (in cm^–1^) is a property of the refractive index (*n*) and dielectric constant (ε) of the solvent, and is described by the Lippert-Mataga [30, 31] equation as below:

1 $${\overline{\upsilon }}_{{\text{A}}} - {\overline{\upsilon }}_{{\text{F}}} = \frac{2}{{{\text{hc}}}}\left( {\frac{{{\upvarepsilon } - 1}}{{2{\upvarepsilon } + 1}} - \frac{{{\text{n}}^{2} - 1}}{{2{\text{n}}^{2} + 1}}} \right)\frac{{\left( {{\upmu }_{{\text{e}}} - {\upmu }_{{\text{g}}} } \right)^{2} }}{{{\text{a}}^{3} }} + {\text{const }}$$

In this equation h = 6.6256 $\times$ 10^–27^ ergs is Planck's constant, c = 2.9979 $\times$ 10^10^ cm/s is the speed of light, and a is the radius of the cavity in which the fluorophore resides. In this equation, the opposite effects of ν_A_ and ν_F_ on the Stokes shift are significant. As the refractive index (n) increases, this energy difference decreases, whereas an increase in ε results in a larger difference between ν_A_ and ν_F_. The refractive index is a rapid frequency response that depends on the motion of electrons in solvent molecules that occur when light is absorbed. In contrast with the refractive index, the dielectric constant is a static and steady feature that depends on the electrons and molecular motions of the solvents’ organization around the excited state. Increasing the refractive index (n) of the ground and excited states is quickly stabilized by the motion of electrons in solvent molecules. This redistribution of electrons reduces the energy difference between ground and excited states. Lippert-Mataga framework, there is no consideration of specific interaction with solvent. Thus, several investigators attempted to extend and modify the Lippert equation. Kawski and co-workers [38–40] obtained a simple quantum mechanical second-order perturbation theory for absorption (ν_A_) and fluorescence (ν_F_) band shifts. By variation of ε and n in solvents, as explained below, functions *f* (*ε*, *n*) and *g*(*n*) refer to Bakhshiev [41] and Kawski‐Chamma‐Viallet [33, 34] relations, respectively. Consequentially the solvent dependent changes for the difference and sum of ν_A_ with ν_F_ have been defined by the following equations:

2 $${\overline{\upsilon }}_{{\text{A}}} - {\overline{\upsilon }}_{{\text{F}}} = m_{1} f\left( {\varepsilon n} \right) + const$$

3 $${\overline{\upsilon }}_{{\text{A}}} + {\overline{\upsilon }}_{{\text{F}}} = - m_{2} \left[ {f\left( {\varepsilon n} \right) + 2g\left( n \right)} \right] + const$$

where:

4 $$f\left( {\varepsilon n} \right) = \frac{{2n^{2} + 1}}{{n^{2} + 2}}\left[ {\frac{{{\upvarepsilon } - 1}}{{2{\upvarepsilon } + 1}} - \frac{{{\text{n}}^{2} - 1}}{{2{\text{n}}^{2} + 1}}} \right]$$

5 $$g\left( n \right) = \frac{3}{2}\left[ {\frac{{n^{4} - 1}}{{\left( {n^{2} + 2} \right)}}} \right]$$

The parameters m_1_ and m_2_ can be determined from absorption and fluorescence band shifts (ῡ_A_ − ῡ_F_) and (ῡ_A_ + ῡ_F_) using the following equations:

6 $$m_{1} = \frac{{2\left( {\mu_{e} - \mu_{g} } \right)^{2} }}{{hca^{3} }}$$

7 $$m_{2} = \frac{{2\left( {\mu_{e}^{2} - \mu_{g}^{2} } \right)}}{{hca^{3} }}$$

where *h* is Planck constant and *c* is the velocity of light; μ_e_ and μ_g_ are the dipole moments in the ground and excited states; and *a* is Onsager radius of the C-dots, which was obtained from the molecular model where the molar volume was calculated by *DFT-B3LYP/6-311*++*G(d,p)* level of theory using Gaussian 0.3 W computer program. The value of the Onsager radius of the C-dots is estimated to be a = 3.97 Å.

**Fig. 3:**
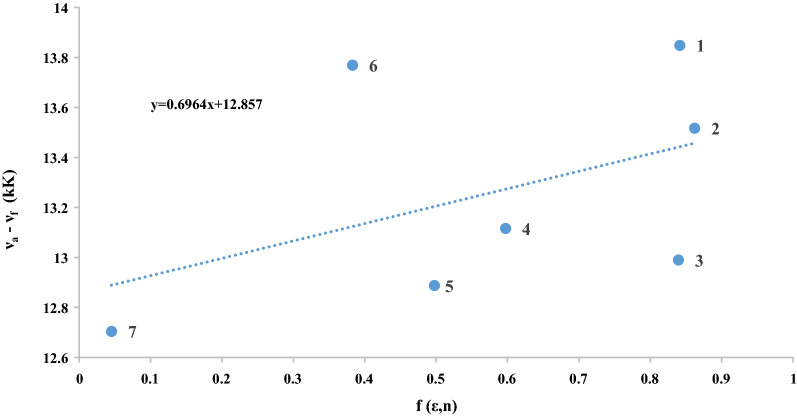
Plot of ν_a_ − ν_f_ (kK) vs. ƒ(ε, n) for C-dots in the aprotic solvents: (1) DMSO, (2) Acetonitrile, (3) DMF, (4) DCM, (5) Acetic acid, (6) Diethyl ether, (7) Dioxane

**Fig. 4:**
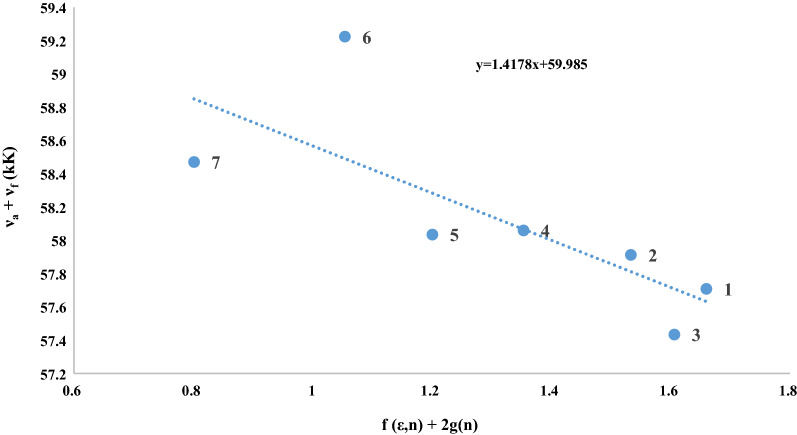
Plot of ν_a_ + ν_f_ (kK) vs. ƒ(ε, n) + 2 g (n) for CDs in the aprotic solvents: (1) DMSO, (2) Acetonitrile, (3) DMF, (4) DCM, (5) Acetic acid, (6) Diethyl ether, (7) Dioxane

Since the slope *m*_*1*_ and *m*_*2*_ from the graph in Figs. 3 and 4, fill in this value in Eqs. 8 and 9 we have:

8 $$\mu_{g} = \frac{{m_{2} - m_{1} }}{2}\sqrt {\frac{{hca^{3} }}{{2m_{1} }}}$$

9 $$\mu_{e} = \frac{{m_{2} + m_{1} }}{2}\sqrt {\frac{{hca^{3} }}{{2m_{1} }}}$$

Thus, the ratio of dipole moments in excited state and ground state is given by:

10 $$\mu_{e} = \frac{{m_{1} + m_{2} }}{{m_{1} - m_{2} }}\mu_{g}$$

The value of Stokes shift varies between 12.70 and 13.84 kK. The values of the Stokes shifts are expressive of the charge transfer transition. The emission of C-dots dispersed in an aprotic solvent such as dioxane (ε = 2.3, α = 0) was recorded at 22.88 kK. While, in the aprotic solvents such as DMSO (ε = 47.27, α = 0), it was registered 21.92 kK. The larger dielectric constants result in a difference in Stokes shift of about 1.14 kK, indicating a charge-transfer transition. The surface external of C-dots can also donate a proton to the aprotic solvent. This interaction influences the emission, notwithstanding the strong hydrogen accepting capability of these solvents from DMSO to dioxane, which is compared by large β Kamlet–Taft parameters (β parameters listed in Table 1) β = 0.76 and 0.37, respectively.

The significant difference in the Stokes shift shows that the structural geometry of the excited state is different from the ground state. Table 2 shows the increase in Stokes shift with increasing aprotic solvent polarity, indicating an increase in dipole moment in the excited state. Spectral shifts (ῡ_A_ − ῡ_F_) and (ῡ_A_ + ῡ_F_) of C-dots in polarity functions *f* (*ε*, *n*) and *f* (*ε*, *n*) + 2* g* (*n*) are illustrated in Figs. 3 and 4. The slopes, intercepts and correlation coefficients of these best-fit lines are listed in Table 2. The slopes *m*_1_ and *m*_2_ of the fitted lines are presented in Table 3, the slopes of Figs. 3 and 4 were estimated to be m_1_ = 696.4 cm^−1^ and m_2_ = 1417.8 cm^−1^. The ground and excited state dipole moment were calculated using Eqs. (8) and (9) respectively, and the values are listed in Table 3. The values found for μ_g_ = 1.077 D, and μ_e_ = 3.157 D, as well as the change in the dipole moments (△μ = μ_g_ − μ_e_) is 1.077 D. The obtained dipole moment shows that the excited state dipole moment (μ_e_) is greater than ground-state dipole moment (μ_g_). It denotes that these C-dots treated as dyes are more polar in the excited states.

**Table 3:** Regression fits to solvatochromic polarity scales for stokes shift of C-dots

Radius 'a' (Å)	μ_g_ (D)	μ_e_ (D)	△μ (D)	μ_e_/μ_g_	m_1_ (cm^−1^)	m_2_ (cm^−1^)
3.97	1.077	3.157	2.08	2.931	696.4	1417.8

### Effect of aprotic solvent on the absorbance and florescence spectra

The typical fluorescence spectra and absorption spectra of C-dots in different aprotic solvents are shown in Figs. 2 and 3, respectively. The emission spectra of C-dots are broad, with shifts depending on the solvents. The large spectral shift is apparent in the fluorescence spectra compared to the absorption spectra. The smaller spectral shift in the absorption spectra than the emission spectra and the higher residence time for fluorescence indicate two phenomena. Firstly, the dipole moment of the excited state is greater than the ground state in all aprotic solvents studied. Secondly, the energy level of the first excited state, *S*_*1*_, is stabilized compared to the ground state, *S*_*0*_, by solvation with increasing the solvent polarity. These phenomena result in a redshift or bathochromic shift of the fluorescence.

The solvent effect was preserved within the outline of the linear solvation energy relationships (LSER) established by Kamlet-Taft [42] multivariate regression, in which each of specific and non-specific interactions has a linear contribution to the total solvation energy of solvent.

11 $$E_{T} = A_{0} + S\pi^{*} + a\alpha + b\beta$$

Here the coefficients π^*^, α and β are the Kamelt-Taft solvatochromic parameters (KAT) which have been developed for scaling the dipolarity/polarizability, hydrogen-bond donor acidity and hydrogen-bond acceptor basicity of solvent, respectively [36, 43, 44]. The *A*_*0*_, *a*, *b* and *s* are regression coefficients, quantity of the sensitivity of E_T_ values to the acidity, basicity and dipolarity/polarizability, respectively. The fit parameters are represented in Table 4. The emission spectra energies were obtained by using Eq. 11.

12 $$E_{T} \left( {\text{kCal.}mol^{ - 1} } \right) = \frac{{hcN_{A} }}{{\lambda_{max} \left( {\text{nm}} \right)}} = \frac{28591}{{\lambda_{max} \left( {\text{nm}} \right)}}$$

**Table 4:** Linear correlations found by plotting E_T_ versus π^*^, α and β according to the KAT equation

Sample	A_0_	s	a	B
C-dots	66.75	− 2.85	0.11	− 1.41

The molar electronic transition energy values, E_T_, of the C-dots in solvents were calculated using Eq. 12. The results related to KAT parameters in fluorescence show that the primary distribution in C-dots is related to polarization interactions. Also, in the as-prepared C-dot, the π^*^ and β sign are negative, indicating the ability to accept hydrogen bonding and polarization. For making the data in Table 4 reasonable and comparable, we have transformed the values in Table 5 into percentage contributions.

**Table 5:** Percentage contribution of solvatochromic parameters in aprotic solvents

KAT parameters	P_π*_ (%)	P_α_ (%)	P_β_ (%)
C-dots	65.21	2.51	32.26

**Fig. 5:**
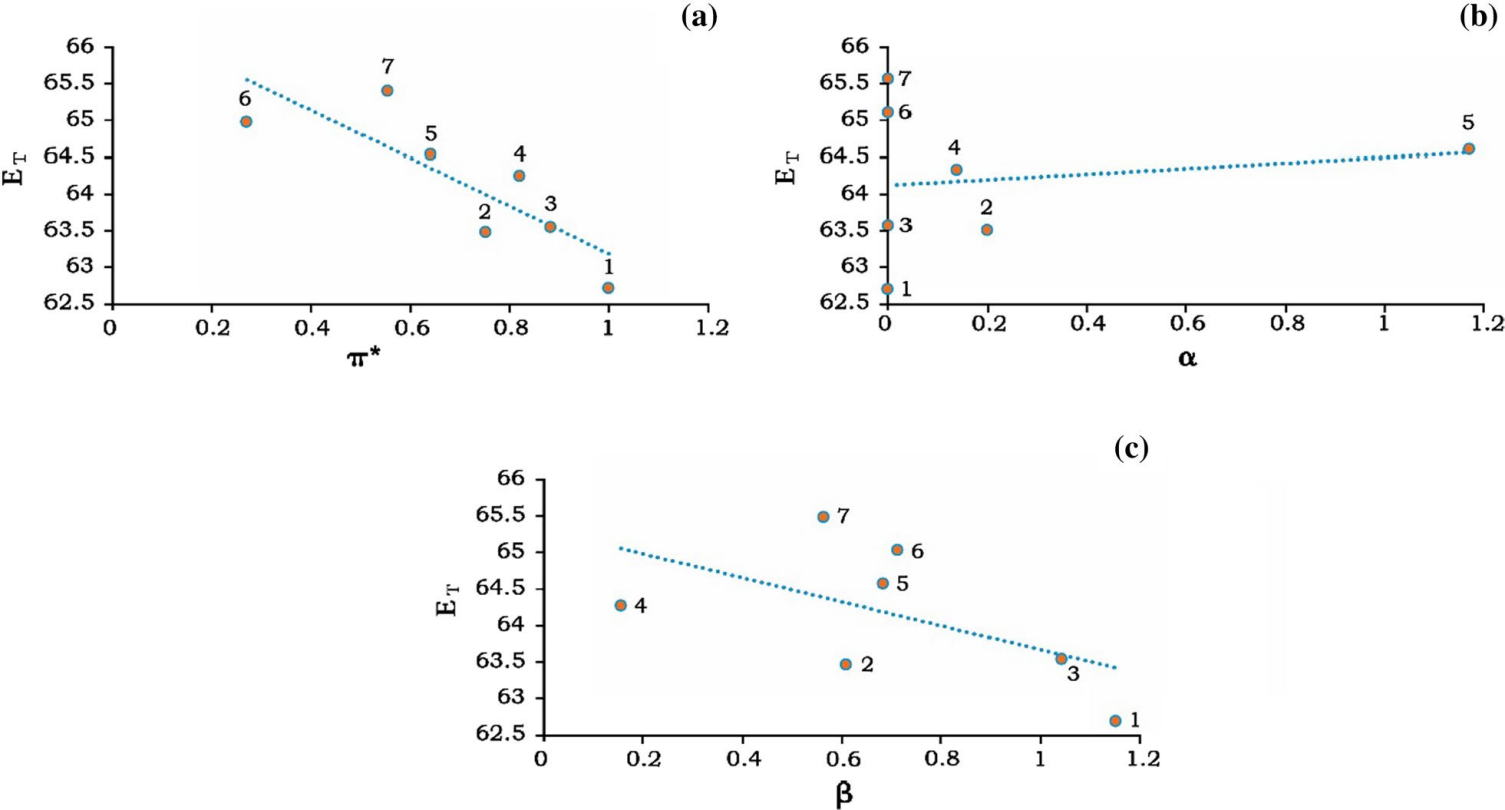
Plot of E_T_ polarity scale with Kamelt-Taft parameters, **a** E_T_ VS. π^*^, **b** E_T_ vs. α and **c** E_T_ VS. β for C-dots in the aprotic solvents: (1) DMSO, (2) Acetonitrile, (3) DMF, (4) DCM, (5) Acetic acid, (6) Diethyl ether, (7) Dioxane

Also, in this study, the focus is on the solvatochromic effect of C-dots with solvents of low dielectric constants. Hence, the π^*^, α and β in aprotic solvents are shown in Fig. 5. As it is indicated in Fig. 5, linear dependence could have resulted from specific and non-specific interactions. The emission maximum E_T_ diagram in π^*^ for the range of aprotic solvents are shown in Fig. 5a. A linear dependence result indicates reasonable linearity with r = 0.7 between E_T_ and π^*^ in all aprotic solvents ranging from DMSO to dioxane. By plotting E_T_ diagrams in terms of α and β, the results showed a poor linear dependence (r = 0.02) between E_T_ and α as well as E_T_ and β (r = 0.3) in the range of aprotic solvents. The reason for this significant deviation from the α parameter can be explained by the use of aprotic solvents that have low acidity, and in most cases, α was zero (Fig. 5a and b).

In contrast to our previous work, if we consider only the aprotic solvents, the results clearly show that the main contribution to solvatochromic is not related to the α scale of solvent HBD acidity and β scale of solvent HBA basicity parameters of solvent. This relationship associates with parameter π^*^, which shows the polarity/polarization of the solvents, and the measure of the stability of a charge or dipole moment with a dielectric effect [45]. The results show that non-specific interaction (dipole–dipole) plays a significant role in solvatochromic of n-π* transition from edge band/edge states of C-dots [44].

The quantum yield of the C-dots dissolved in aprotic solvents was defined at an excitation wavelength of 350 nm using the following equation (Eq. 13)

13 $${\text{Q}}_{{{\text{CDs}}}} {\text{ = Q}}_{{\text{R}}} \cdot \frac{{{\text{I}}_{{{\text{CDs}}}} }}{{{\text{I}}_{{\text{R}}} }} \cdot { }\frac{{{\text{A}}_{{\text{I}}} }}{{{\text{A}}_{{{\text{CDs}}}} }} \cdot { }\frac{{{\upeta }_{{{\text{CDs}}}}^{{2}} }}{{{\upeta }_{{\text{R}}}^{{2}} }}$$

where *Q* is the quantum yield, *I* is the intensity of fluorescence spectra, *A* is the absorbance at the excitation wavelength, and η is the refractive index (1.33) of the solvent using quinine sulfate (quantum yield 0.5) in 0.1 M H_2_SO_4_ solution as the reference [46, 47]. The subscripts ‘CDs’ stands for carbon dots and ‘R’ for the reference are used in this equation. while the C-dots was dissolved in water (η = 1.33), as the reference solution to keep their absorption at minimum ˂ 0.05 by comparing the integrated fluorescence intensities using the Eq. 13 at excitation wavelength of 350 nm and the quantum yield was determinded. The quantum yield results calculated for C-dots are given in Table 1. According to the results, the highest amount of quantum yield among the studied aprotic solvents was obtained in diethyl ether (with a quantum yield of 0.53). This high quantum yield value can be attributed to the minimum value of AN in diethyl ether, among other aprotic solvents studied. To further explain this, it is assumed that the combination of other factors, such as the carbon core domain, affects emissions. However, solvatochromism deals with the effects of surface groups, and in the case of organic solvents, the effect of specific interactions such as hydrogen bond donors was minimal. Henceforth, if we limit our attention to the electron-donating and accepting characters of the constituents, the functional groups on the surface of C-dots containing –NH_2_ and –OH groups act as electron donors in their excited states. Thus, the surface energy traps are stabilized next to less polar solvents with lower acceptor number (AN) value and eventually promoted emission efficiency results [29, 49]. At the practical level, further studies and research on the optical properties of C-dots, including quantum yield, are conserved in a wide range of solvents, and some of these solvents are potentially useful in biological applications. As the results illustrated in Table 1 the fluorescence efficiency in some of the solvent are significant. Nonetheless, they show no clear trend. Consistent with our previous study [35], it was shown that the solvent with greater AN (the higher electron-accepting character can be seen from their α values) was a more effective quencher.

## Conclusions

In summary, in this research work, we dissolved C-dots in various aprotic solvents to explore the specific solvation effects. A different factor is expressed on C-dots solvent-soluble interaction is generally controlled by the polarizability and basicity parameters. Studies were performed to calculate the dipole moment. The results showed that the excited state dipole moment is higher than the ground state. The results related to KAT parameters in fluorescence indicates the occurance of a bathochromic shift of the fluorescence.

One of the grand challenges in studying C-dots for biological applications and bio-imaging is to increase the duration and intensity of C-dots fluorescence. Steps for more efficient use of C-dots in this field prompted us to evaluate quantum yields, and the resulted values indicated significant improvements of C-dot’s quantum yields in some aprotic solvents.

## Data Availability

All datasets generated or analyzed during the current study are included in this published article.

## Abbreviations

C-dots: Carbon dots
PL: Photoluminescence
KAT: Kamelt-Taft
NaOH: Sodium hydroxide
DMSO: Dimethyl sulfoxide
DMF: Dimethylformamide
DCM: Dichloromethane
FE-SEM: Field emission scanning electron microscopy
FT-IR: Fourier transform infrared
LSER: Linear solvation energy relationships
HBA: Hydrogen bond acceptor
HBD: Hydrogen bond donor
